# Occupational low back pain prevention capacity of nurses in China: A multicenter cross-sectional study

**DOI:** 10.3389/fpubh.2023.1103325

**Published:** 2023-03-16

**Authors:** Qianru Liu, Xue Liu, Huijing Lin, Yu Sun, Li Geng, Yongli Lyu, Mengna Wang

**Affiliations:** ^1^Union Hospital, Tongji Medical College, Huazhong University of Science and Technology, Wuhan, China; ^2^School of Nursing, Tongji Medical College, Huazhong University of Science and Technology, Wuhan, China; ^3^School of Nursing, Wuhan University, Wuhan, China

**Keywords:** nurse, occupational low back pain, prevention capacity, influencing factors, multicenter cross-sectional study

## Abstract

**Introduction:**

Nurses have a high prevalence of occupational low back pain, especially since the outbreak of the COVID-19 pandemic, which has increased the nurses' workloads. It has brought a huge burden on nurses and their professional development. Nurses' occupational low back pain prevention capacity is the logical starting point and core of interventions to prevent its occurrence. To date, there is no study investigating it with a scientific scale. Therefore, a multicenter cross-sectional study was conducted to explore the current status of nurses' capacity in occupational low back pain prevention and its influencing factors in China.

**Methods:**

Using a two-stage, purposive and convenience mixed sampling method, 1331 nurses from 8 hospitals across 5 provinces (Hubei, Zhejiang, Shandong, Henan, and Sichuan) in the southern, western, northern, and central areas of mainland China were involved in this study. The demographic questionnaire and occupational low back pain prevention behavior questionnaire were used for data collection. The descriptive analysis, univariate analysis, and multiple stepwise linear regression were used for data analysis.

**Results:**

The results showed that the occupational low back pain prevention behavior questionnaire score was 89.00 (80.00, 103.00) [M (Q1, Q3)], which indicated that nurses' ability was at a moderate level. Participation in prevention training before, perceived stress at work, and working hours per week were predictors for nurses' occupational low back pain prevention capacity.

**Discussion:**

To improve nurses' prevention ability, nursing managers should organize various training programs, strengthen regulations to reduce nurses' workload and stress, provide a healthy workplace, and offer incentives to motivate nurses.

## 1. Introduction

Occupational low back pain, defined as pain or discomfort from the lower edge of the 12th rib of the pelvis caused by occupational causes, has become one of the three primary occupational health concerns (1). Compared to other professions, nurses have a higher prevalence of occupational low back pain given the suffering of chronic lumbar strain and elevated spinal pressure at work (2), especially since the outbreak of the COVID-19 pandemic, which has increased the nurses' workloads (3). According to the findings of a systematic review, the prevalence of occupational low back pain among nurses ranges from 50 to 80% globally (4). Additionally, more than 70% of nurses repeatedly experience low back discomfort (5). The high prevalence indicates that occupational low back pain has become a non-negligible disease affecting the health of nurses.

Occupational low back pain has brought a huge burden on nurses and their professional development. Occupational low back pain endangers the safety of nurses and patients since it can decrease nurses' working ability and hamper their mobility (6, 7). Due to their sense of responsibility and empathy, many nurses with low back pain insist on working (8). However, the intricacy of the work in turn may exacerbate their low back pain (9). The relationship between nurses' absent work days with occupational low back pain has been extensively documented (10, 11), posing a substantial challenge to nursing resources and professional stability. In addition, nurses' mental wellbeing, quality of life, and financial situation may be impacted by low back pain (12, 13). Therefore, more attention needs to be paid to improving the occupational low back pain and the health status of nurses.

Prevention is an indispensable step to stopping harmful health behaviors at an early stage. Preventing low back pain is critical since it could improve the productivity, job satisfaction, and job safety of nurses (14). Many interventional measures, such as training, exercise, and organizational interventions, have been applied in preventing occupational low back pain (15, 16). For example, Sihawong et al. (17) found that muscle stretching and endurance exercise could prevent low back pain occurrence. Nguyen et al. (18) indicated that a mixed training program of knowledge education and physical exercise effectively prevents low back pain prevalence. Nevertheless, many systematic reviews (5, 11, 19) have concluded that the effectiveness of these interventions in occupational low back pain prevention remains highly controversial. Xu (20) has identified competence as the logical starting point and core of interventions. It has been demonstrated that the competency-based approach could enhance the effectiveness and efficiency of interventions (21). Therefore, it is crucial to investigate nurses' ability to prevent occupational low back pain, which could identify risk factors affecting their prevention and develop more targeted intervention programs (22).

**Figure 1:**
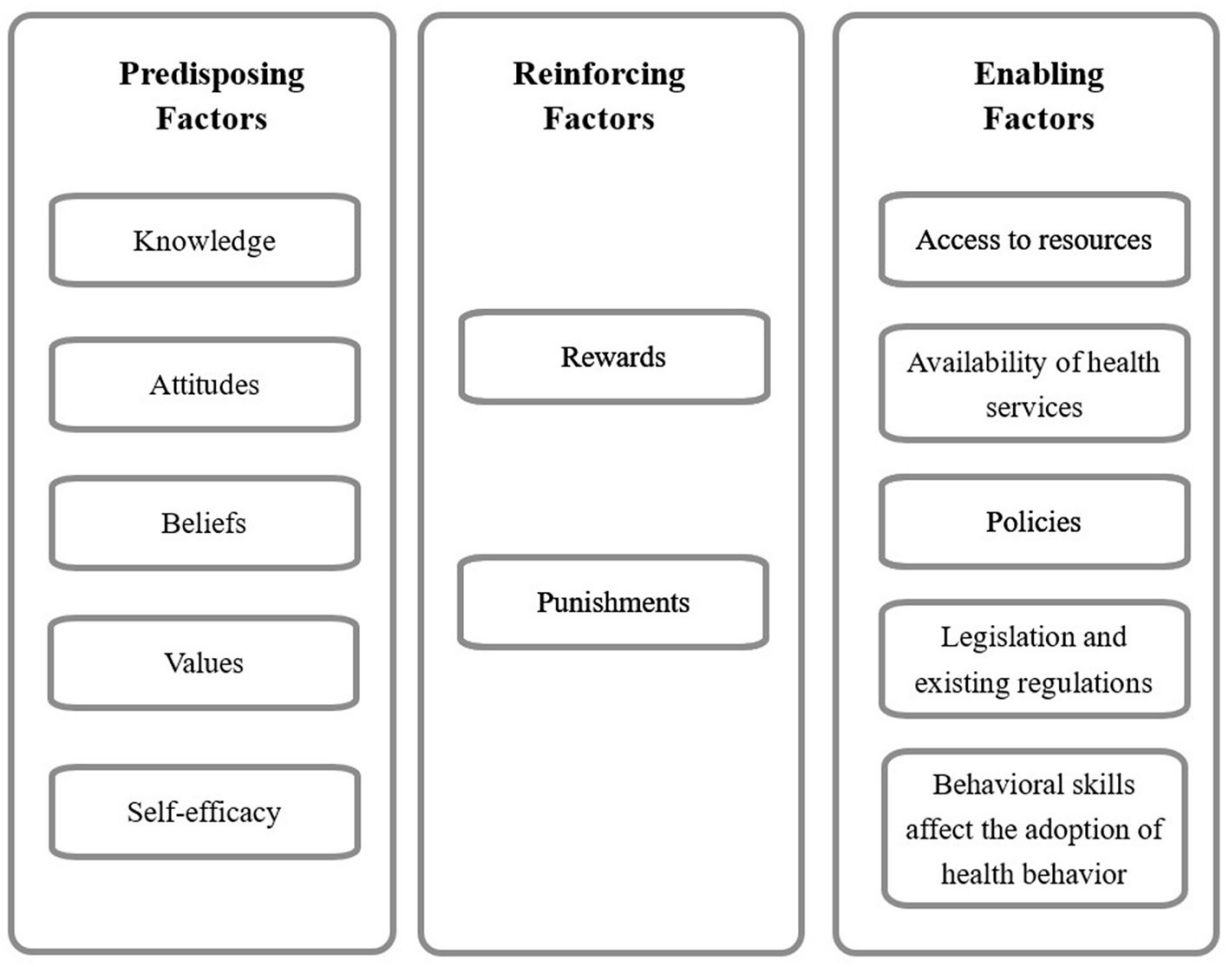
Factors of occupational low back pain prevention ability.

Based on the Predisposing, Reinforcing, and Enabling Constructs in Educational Diagnosis and Evaluation-Policy, Regulatory, and Organizational Constructs in Educational and Environmental Development (PRECEDE-PROCEED) model, Kazemi et al. (23) developed the occupational low back pain prevention behavior questionnaire to comprehensively and accurately assess nurses' ability to prevent occupational low back pain, involving predisposing, reinforcing, and enabling factors of the ability, which serves as the cornerstone for providing appropriate health promotion programs and strategies (Figure 1).

To our knowledge, there has been no study investigating nurses' ability to prevent occupational low back pain utilizing a scientific scale, limiting the value of assessing competency in establishing health promotion programs. Therefore, this study attempted to address three scientific questions: (1) what is the ability of nurses to prevent occupational low back pain? (2) which factors affect nurses' ability? (3) based on the findings of this study, what can nursing managers do to improve, promote, and strengthen nurses' competencies?

## 2. Methods

### 2.1. Study design

This study was approved by the ethics committee of Tongji Medical College, Huazhong University of Science and Technology (registration number: 2022-S111). A multicenter cross-sectional study was conducted from August 2022 to October 2022.

### 2.2. Participants

A two-stage sampling method was used. First, the availability of participants was considered to achieve a homogeneous regional distribution of the samples. Five provinces (Hubei, Zhejiang, Shandong, Henan, and Sichuan) in the eastern, western, southern, and central areas of mainland China were selected using the purposive sampling method. Second, the convenience sampling method was used to select eight hospitals from the above areas. Registered nurses from eight hospitals who had obtained nursing certificates were enrolled. Nurses with physiological low back pain (caused by menstruation or during pregnancy, etc.) or pathological low back pain (caused by diseases such as tumors, previous back surgery, compulsory spondylitis, scoliosis, etc.) were excluded (24). Whether nurses have physical low back pain was based on their self-described condition, and pathological low back pain was based on the physician's diagnosis.

The empirical method of sample estimation for analytical studies was used to calculate the sample size for this study, which means that the sample size should be at least 10–15 times the number of variables analyzed. A total of 22 variables were analyzed in this study. Considering a 20% of sample attrition rate, the minimum sample size required for this study was 396.

### 2.3. Instruments

#### 2.3.1. Demographic questionnaire

General demographic characteristics (including age, body mass index (BMI), gender, marital status, education level, and parity), work-related information (including title, working years, working department, working hours per week, night shifts per week, the daily working step, perceived stress at work), occupational low back pain history, and whether they have participated in prevention training were collected as demographic variables.

In addition, perceived stress at work is defined as confusion or threat caused by occupational factors (e.g., high-intensity workloads), which could cause tension and discomfort physically and mentally (25). Various programs aiming to prevent occupational low back pain, including knowledge education, nursing posture training, and functional exercise, are considered prevention training.

#### 2.3.2. Occupational low back pain prevention behavior questionnaire

The Chinese version of the occupational low back pain prevention behavior questionnaire (OLBPPBQ) was used to assess nurses' capacity to prevent occupational low back pain (26). The Cronbach's α was 0.91, indicating that it can be adopted as a useful assessment tool. It contains 29 items in six dimensions: knowledge, attitude, self-efficacy, reinforcing factors, enabling factors, and behavior. Nurses' knowledge, beliefs, motivation, and behavioral skills to prevent occupational low back pain were assessed through the “knowledge”, “attitude”, and “self-efficacy” dimensions. The “reinforcing factors” dimension was used to assess rewards that help nurses prevent low back pain (e.g., providing gym cards, health education programs, etc.). The “enabling factors” dimension was used to assess the supportive workplace environment (e.g., administrative policy, resources) that assists occupational low back pain prevention. Nurses' previous behavioral status of occupational low back pain prevention was assessed by the “behavior” dimension. All items were scored using a 5-point Likert scale (1 = never, 5 = always) except for items in the knowledge dimension where correct answers were scored 5 and incorrect answers or “I don't know” were scored 1. The questionnaire scores range from 29 to 145. Higher scores indicated that nurses had a better ability to prevent occupational low back pain. The Cronbach's α in this study was 0.93.

### 2.4. Data collection

Data collection in this study was implemented online. The link to the questionnaire (www.wjx.cn/vm/YyWBKVa.aspx) was formed through “Questionnaire Star,” a professional online questionnaire collection platform. After obtaining permission, nursing managers from each hospital were invited to assist in the data collection. The link to the questionnaire was sent to those nursing managers, who then delivered it to the nurses. The purpose of the survey and the requirements for completion were explained on the guide page, and anonymity was guaranteed. Nurses were informed that they could withdraw from the survey during the completion process. Nurses who completed the intact questionnaire were considered to have consented to participate in this study.

To avoid any influence on the knowledge dimension, the question about occupational low back pain history was asked as the last question, with a prefix explaining the definition of occupational low back pain. In addition, the daily working step was calculated based on professional step-counting software (e. g. WeChat Sports) to ensure accuracy.

### 2.5. Data analysis

All statistical analyses were conducted in IBM SPSS version 27.0. Descriptive analysis was used to describe the characteristics of participants. The data fitting normal distribution were expressed using mean (standard deviation, SD); otherwise, median and quartiles [M (Q1, Q3)] were used. The *t*-test, *F*-test, Mann–Whitney *U*-test, and Kruskal–Wallis *H*-test were used to compare between-group differences, if appropriate. Multiple linear stepwise regression analysis was used to identify factors influencing nurses' occupational low back pain prevention ability. The *P*-value < 0.05 was considered statistically significant.

## 3. Results

A total of 1,600 questionnaires were distributed, and 1,416 questionnaires were returned, with a response rate of 88.50%. After excluding questionnaires with regular answers and invalid data, 1,331 valid questionnaires were used for analysis, with an effective response rate of 94.00%.

### 3.1. Participants' demographics

The participants' demographics are shown in Table 1. Participants' age in this study ranged from 20 to 60 years old, mostly 30–34 years(27.50%) with a mean (SD) age of 31.48 (7.19) years old. Majority of participants were female (95.27%), with BMI ranging from 18.50 to 23.9 (69.95%), having children (54.85%), married (59.73%), and with a bachelor's degree (88.28%). They mostly had the title of nurse practitioner (38.77%), and worked < 5 years (30.20%). They were required to work for mostly 40-45 hours (64.24%) and 1–2 night shifts (45.15%) per week, with an average of 10000-199999 steps daily (78.44%). In addition, more than half of them felt stressed at work (79.34%), had a history of low back pain (67.62%), and participated in prevention training previously (83.02%).

**Table 1:** Demographics of participants (*N* = 1331).

**Variables**		***N* (%)**
Age	< 25	232 (17.43%)
	25–29	365 (27.42%)
	30–34	366 (27.50%)
	35–39	163 (12.25%)
	≥40	205 (15.40%)
BMI	< 18.5	167 (12.55%)
	18.50–23.9	927 (69.65%)
	24-27.9	192 (14.43%)
	≥28	45 (3.38%)
Gender	Male	63 (4.73%)
	Female	1268 (95.27%)
Marital status	Unmarried	518 (38.92%)
	Married	795 (59.73%)
	Divorced/widowed	18 (1.35%)
Education level	Junior college	112 (8.41%)
	Bachelor degree	1175 (88.28%)
	Master and above	44 (3.31%)
Parity	≥1	730 (54.85%)
	0	601 (45.15%)
Title	Nurse	297 (22.31%)
	Nurse practitioner	516 (38.77%)
	Nurse in charge	506 (38.02%)
	Deputy chief/chief nurse	12 (0.90%)
Working years	0~	402 (30.20%)
	5~	354 (26.60%)
	10~	306 (22.99%)
	15~	120 (9.02%)
	20~	149 (11.19%)
Working department	Internal medicine	317 (23.82%)
	Surgery	412 (30.95%)
	Pediatrics	63 (4.73%)
	Obstetrics/Gynecology	199 (14.95%)
	Emergency/outpatient	99 (7.44%)
	Intensive care unit	59 (4.43%)
	Operating room	84 (6.31)
	Others	98 (7.36%)
Working hours per week	< 40	45 (3.38%)
	40~	855 (64.24%)
	45~	232 (17.43%)
	50~	199 (14.95%)
Night shifts per week	≤1	571 (42.90%)
	≤2	650 (48.84%)
	≤3	90 (6.76%)
	≤4	20 (1.50%)
Daily working step	< 10000	222 (16.88%)
	10000–19999	1,044 (78.44%)
	20000–29999	51 (3.83%)
	≥30000	14 (1.05%)
Stress at work	Yes	1,056 (79.34%)
	No	275 (20.66%)
Occupational low back pain history	Yes	900 (67.62%)
	No	431 (32.38%)
Participation in prevention training before	Yes	1105 (83.02%)
	No	226 (16.98%)

BMI is the body mass index.

### 3.2. Occupational low back pain prevention capability

The median overall OLBPPBQ score was 89 (80, 103), indicating that the ability of nurses to prevent occupational low back pain was moderate. The median scores of the knowledge, attitude, self-efficacy, reinforcing factors, enabling factors, and behavior dimensions were 12 (8, 12), 20 (17, 20), 15 (12, 18), 15 (5, 12), 21 (18, 24), and 9 (8, 12), respectively. Table 2 shows the scores of each item of OLBPPBQ. In particular, the items of the knowledge dimension are expressed as correct rates.

**Table 2:** The score of each item of the occupational low back pain prevention behavior questionnaire.

**Dimension**	**Number**	**Item**	**Score [M (Q1, Q3)]/ Correct rate**
Knowledge (K)	K1	What is occupational low back pain?	26.22%
	K2	Which item can increase occupational low back pain?	2.78%
	K3	What causes spinal damage?	78.06%
	K4	What is the treatment for occupational low back pain?	70.92%
Attitude (A)	A1	If I follow the back pain prevention practices during work, I can do my job in ease without delay.	4.00 (3.00,4.00)
	A2	Establishing a supportive relationship with colleagues in the workplace helps to reduce stress and prevent low back pain.	4.00 (3.00,4.00)
	A3	Training on low back pain prevention behaviors in nurses is a necessity.	4.00 (4.00,5.00)
	A4	Doing preventive behaviors related to low back pain is one of my priorities.	4.00 (3.00,4.00)
	A5	Back pain reduction in workplace might lead to reduction in absenteeism.	4.00 (3.00,4.00)
Self-efficacy (S)	S1	I can do the prevention behavior of low back pain in my workplace.	3.00 (2.00,4.00)
	S2	I can change the position in different situations to prevent low back pain (from standing to sitting and *vice versa*).	3.00 (3.00,4.00)
	S3	I have the skill of doing low back pain prevention behaviors.	3.00 (2.00,3.00)
	S4	I can manage stressful conditions in the workplace by working out a supportive relationship.	3.00 (3.00,4.00)
	S5	Despite many works, I can devote some times for doing low back pain prevention behaviors.	3.00 (2.00,3.00)
Reinforcing factors (R)	R1	My colleagues encourage me to keep my spine healthy at work.	3.00 (2.00,4.00)
	R2	Providing a low-cost or discounted gym membership card and existence of bodybuilding at the workplace are encouraging factors in the prevention of low back pain.	3.00 (2.00,4.00)
	R3	Attractive health educational programs for low back pain prevention in the workplace encourage me to do these behaviors.	3.00 (2.00,4.00)
	R4	Getting a certificate and receiving rewards due to participation in a training program to prevent low back pain in the workplace encourages me to do these behaviors.	3.00 (2.00,4.00)
	R5	Feeling good after having low back pain prevention behaviors will keep me from risky behaviors.	3.00 (2.00,4.00)
Enabling factors (E)	E1	The volume of my work is such that I have the opportunity to prevent myself from low back pain.	3.00 (2.00,4.00)
	E2	The rules and regulations at my workplace support the prevention of low back pain.	3.00 (2.00,4.00)
	E3	At my workplace, the presence of a sports training area helps prevent back pain.	3.00 (2.00,4.00)
	E4	Between activities in my workplace, having equipment such as a chair for sitting and a footstool next to the bed of the operating room for intermittent breaks helps prevent back pain.	3.00 (3.00,4.00)
	E5	At my workplace, the educational needs of the nursing staff are planned and implemented in the nursing department.	3.00 (3.00,4.00)
	E6	At my workplace, in-person training on the prevention of low back pain treatment is applicable.	3.00 (3.00,4.00)
	E7	At my workplace, training *via* social media on low back pain prevention behaviors is feasible.	3.00 (3.00,4.00)
Behavior (B)	B1	During my work, I maintain the correct position of the spine, such as moving legs in a standing position and lifting heavy objects with the help of leg muscles.	3.00 (3.00,4.00)
	B2	I carry out targeted exercises for strengthening the muscles of the waist, abdomen, and thighs.	3.00 (2.00,4.00)
	B3	By deep breathing and relaxing, I manage stress in my workplace.	3.00 (3.00,4.00)

M is the median; Q1 is the first quartile; Q3 is the third quartile.

### 3.3. The influencing factors of nurses' capacity to prevent occupational low back pain

The between-group differences in OLBPPBQ scores of demographic variables are shown in Table 3. The results showed that the OLBPPBQ scores were statistically different in age, gender, title, working years, working hours per week, perceived stress at work, history of occupational low back pain, and participation in prevention training before.

**Table 3:** Between-group differences in OLBPPBQ scores of demographic variables.

**Variables**		**OLBPPBQ score**		**Z/H**	***P***
		**M**	**Q1, Q3**		
Age	< 25	92.00	83.00, 105.00	12.17	0.02
	25–29	90.00	81.00, 105.00		
	30–34	89.00	79.00, 104.00		
	35–39	87.00	78.00, 102.00		
	≥40	87.00	79.50, 98.00		
BMI	< 18.5	88.00	78.00, 105.00	0.72	0.87
	18.50–23.9	90.00	81.00, 103.00		
	24–27.9	89.00	81.00, 103.00		
	≥28	88.00	81.00, 112.50		
Gender	Male	92.00	85.00,104.00	−2.19	0.03
	Female	89.00	80.00,103.00		
Marital status	Married	90.00	81.00, 105.00	1.36	0.51
	Unmarried	89.00	80.00, 117.00		
	Divorced/widowed	88.50	82.00, 101.00		
Education level	Junior college	87.50	80.00, 106.75	3.38	0.19
	Bachelor degree	89.00	80.00, 103.00		
	Master and above	94.50	86.25, 106.00		
Parity	≥1	88.00	79.75, 102.00	−1.96	0.05
	0	90.00	82.00, 105.00		
Title	Nurse	92.00	82.50, 105.00	11.52	0.01
	Nurse practitioner	90.00	80.00, 104.00		
	Nurse in charge	87.00	79.00, 102.00		
	Deputy chief/chief nurse	92.50	84.25, 101.50		
Working years	0~	92.00	82.00, 106.00	11.74	0.02
	5~	90.00	79.75, 101.00		
	10~	87.00	79.00, 103.25		
	15~	89.00	80.00, 99.00		
	20~	88.00	79.00, 101.50		
Working department	Internal medicine	89.00	80.00, 103.00	8.29	0.31
	Surgery	91.00	81.25, 105.00		
	Pediatrics	89.00	79.00, 100.00		
	Obstetrics/Gynecology	89.00	81.00, 104.00		
	Emergency/outpatient	86.00	79.00, 101.00		
	Intensive care unit	95.00	82.00, 105.00		
	Operating room	88.00	77.25, 98.75		
	Others	88.50	78.00, 108.00		
Working hours per week	< 40	86.00	80.50, 109.50	7.81	0.04
	40~	91.00	81.00, 104.00		
	45~	88.50	80.25, 101.00		
	50~	87.00	76.00, 101.00		
Night shifts per week	≤1	89.00	82.00, 103.00	4.01	0.26
	≤2	90.00	80.00, 104.00		
	≤3	86.00	77.75, 104.00		
	≤4	83.50	78.25, 103.00		
Daily working step	< 10000	90.00	82.00, 104.25	4.63	0.20
	10000–19999	89.00	69.00, 104.00		
	20000–29999	84.00	78.00, 99.00		
	≥30000	86.50	77.50, 92.00		
Perceived stress at work	Yes	88.00	85.00, 109.00	−6.09	< 0.01
	No	95.00	79.00, 101.00		
Occupational low back pain history	Yes	88.00	79.00, 102.00	−3.64	< 0.01
	No	92.00	83.00, 106.00		
Participation in prevention training before	Yes	92.00	83.00, 106.00	−13.852	< 0.01
	No	77.00	66.00, 87.00		

OLBPPBQ is the occupational low back pain prevention behavior questionnaire; M is the median; Q1 is the first quartile; Q3 is the third quartile; BMI is the body mass index.

Multiple stepwise linear regression analysis was conducted, with these variables as independent variables and OLBPPBQ scores as dependent variables (Table 4). Results indicated that participation in prevention training before, perceived stress at work, and working hours per week entered the regression model (*F* = 96.438, *p* < 0.001), which explained 17.7% of the variance in OLBPPBQ scores (adjusted R^2^ = 0.177). The standardized regression coefficients (Beta) revealed that the capacity to prevent occupational low back pain was enhanced for nurses who had participated in prevention training and were not stressed at work. In addition, the ability declined as the working hours per week increased.

**Table 4:** Multiple linear stepwise regression analysis for influencing factors of nurses' occupational low back pain prevention capability.

**Item**	***b***	**SE–b**	**Beta**	***t***	***p***	**95.0% CI**	
						**Lower**	**Upper**
Constant	130.439	2.82		46.263	< 0.001	124.908	135.971
Participation in prevention training before	−18.956	1.232	−0.383	−15.38	< 0.001	−21.374	−16.538
Perceived stress at work	−6.893	1.147	−0.15	−6.009	< 0.001	−9.144	−4.643
Working hours per week	−1.645	0.593	−0.069	−2.774	0.006	−2.809	−0.482

b is the unstandardized regression coefficient; SE-b is the standard error; Beta is the standardized regression coefficient; CI is the confidence coefficient.

In this survey, 86.7% of nurses with a history of low back pain had attended low back pain training, and 13.3% had not participated in low back pain training. Nurses with a history of low back pain were divided into two groups based on whether they had participated in prevention training. The scores of OLBPPBQ were compared between these two groups (Table 5). The results showed that the OLBPPBQ scores of nurses who had participated in prevention training were higher.

**Table 5:** Between-group differences in OLBPPBQ scores of nurses with a history of occupational low back pain.

**Participation in prevention training before**	**OLBPPBQ scores [M (Q1, Q3)]**	***Z***	***P***
Yes	91.00 (82.00, 105.00)	−11.877	< 0.001
No	76.00 (64.00, 86.00)		

OLBPPBQ is the occupational low back pain prevention behavior questionnaire; M is the median; Q1 is the first quartile; Q3 is the third quartile.

## 4. Discussion

This multicenter cross-sectional study was conducted in eight tertiary hospitals from five provinces in the east, west, south, and central areas of mainland China. This is the first study to explore the current status of Chinese nurses' ability in occupational low back pain prevention and its influencing factors.

### 4.1. The current status of nurses' capacity to prevent occupational low back pain

In this study, the ability of nurses to prevent occupational low back pain was modest, as indicated by the median OLBPPBQ total score of 89 (80, 103). Compared to the nurses' ability to avoid other occupational diseases, such as varicose veins (27) and dermatitis (28), their ability to prevent low back pain is relatively low.

The attitude dimension got a higher average score than the other five dimensions, which indicated that nurses in this multidisciplinary study had positive attitudes toward preventing low back pain, similar to the study of rehabilitation nurses (29), but showed more positive attitudes than ICU nurses (30). The knowledge-attitude-behavior (KAB) model suggests that attitude is the motivation, and knowledge is the basis for behavioral change (31). However, the low correct rates of K1 (What is occupational low back pain? 26.22%) and K2 (Which item can increase occupational low back pain? 2.78%) indicated that only a minority of nurses had accurate comprehension of the concept and risk factors for occupational low back pain. Therefore, despite nurses' positive attitudes toward preventing occupational low back pain, the behavior score was at a moderate level. In addition, heavy workloads due to an unequal nurse-patient ratio make nurses have limited time for low back pain prevention behaviors, such as exercise and rest. In our study, most of the nurses stated the necessity of training on low back pain prevention behavior in nurses (A3: Training on low back pain prevention behaviors in nurses is a necessity), suggesting a routine training program, such as theoretical lectures and operational demonstrations, is needed to improve knowledge of body mechanics and body posture at work, which ultimately contributes to the successful practice of low back pain prevention. Meanwhile, a knowledge-learning library can be created to offer learning and training resources online.

Self-efficacy serves as a protective factor against workplace misconduct (32, 33). In this study, nurses' self-efficacy for preventing occupational low back pain was at a moderate level, and the aspects of skills (S3: I have the skill of doing low back pain prevention behaviors.) and times (S5: Despite many works, I can devote some times for doing low back pain prevention behaviors) showed low levels, which may be due to the lack of knowledge and training on how to prevent low back pain. It has been demonstrated that poor knowledge is associated with poor risk perception of low back pain among nurses (34), which has a negative impact on low back pain prevention behaviors (35). Gohner et al. (36) found that using a cognitive-behavioral training program could strengthen severity perceptions and reduce barrier perceptions, thus overcoming the intention-behavior gap and strengthening regular exercises to prevent chronic back pain. Therefore, a cognitive-behavioral training program could be set up to help nurses build positive cognition (37), which is beneficial for self-management behaviors useful for the prevention of low back pain.

The reinforcing factors dimension and the enabling factors dimension assessed nurses' ability to prevent occupational low back pain from workplace factors, which is critical for nurses to conduct health-promoting behaviors and to enhance long-term capacity (38, 39). The results showed that scores of both dimensions were at moderate levels. In particular, the above-average scores of E4 (Providing a low-cost or discounted gym membership card and existence of bodybuilding at the workplace are encouraging factors in the prevention of low back pain.), E5 (At my workplace, the educational needs of the nursing staff are planned and implemented in the nursing department.), E6 (At my workplace, in-person training on the prevention of low back pain treatment is applicable.), and E7 (At my workplace, training *via* social media on low back pain prevention behaviors is feasible.) indicated that a workplace with enough equipment (e.g. chairs and footstools) and resources of education and training is useful to prevent occupational low back pain (40). Meanwhile, awards (R2: Providing a low-cost or discounted gym membership card and existence of bodybuilding at the workplace are encouraging factors in the prevention of low back pain, R3: Attractive health educational programs for low back pain prevention in the workplace encourage me to do these behaviors, and R4: Getting a certificate and receiving rewards due to participation in a training program to prevent low back pain in the workplace encourages me to do these behaviors) and peer support (R1: My colleagues encourage me to keep my spine healthy at work) positively affect health behaviors and outcomes (41), which were at moderate levels in this study. Therefore, incentives can motivate nurses to engage in preventive low back pain behaviors and should be provided properly.

### 4.2. The influencing factors of nurses' capacity to prevent occupational low back pain

This study found that participation in low back pain prevention training was a predictor of occupational low back pain prevention capabilities and nurses who had attended the training before had better prevention capabilities. Substantial evidence has demonstrated that training (e.g., physical training, theoretical training, and cognitive-behavioral training), as the principal method for preventing and reducing occupational low back pain in healthcare institutions (42), contributes to the enhanced prevention ability by improving low back pain prevention knowledge, individual physical abilities, work practice skills, and work attitudes (43). Therefore, various types of training programs should also be provided.

It has been observed that working hours per week and perceived stress at work were also predictors of nurses' ability to occupational low back pain prevention, and nurses with perceived stress and long working hours had a poorer ability. The excessive workload is the primary driver of work stress for Chinese nurses (44). High-intensity workloads and work stress discourage nurses from participating in health education programs or training for occupational low back pain prevention and obstruct nurses from adopting low back pain prevention behaviors. Therefore, nursing managers should take measures to allocate human resources rationally and to reduce the workload of nurses. As recommended by the National Health Commission of the People's Republic of China (45), a flexible schedule of nursing shifts should be introduced to boost the nursing workforce, which has also been demonstrated to enhance nurses' retention (46). Meanwhile, it is essential to implement mental and physical relaxation to reduce nurses' occupational stress (47).

This study also found that among nurses with a history of low back pain, those who had attended prevention training had a higher ability to prevent occupational low back pain than those who had not. Like Denis et al. (48) opinion, this finding indicated that targeted training is positive for nurses with recurrence and chronicity risk of occupational low back pain. Low back pain often recurs among nurses (5). Undoubtedly, it is essential to prevent or delay the progression of low back pain (secondary prevention), as well as to reduce the intensity of low back pain and prevent complications and disability (tertiary prevention) (49). Consequently, training programs for nurses with occupational low back pain history, such as ergonomics training and education (50), should be adopted, thus improving secondary and tertiary prevention.

### 4.3. Limitations

This study had several limitations. First, due to time and financial restrictions, we have only chosen five provinces from the southern, western, eastern, and central areas of China, which limited the generalizability. Future studies should select more provinces and regions to robust the study findings. Second, the cross-sectional study could only assess the ability of nurses to prevent low back pain at a specific time. It is expected that future researchers could develop more intervention strategies and monitor the improvements in methods. Third, the nurses' occupational low back pain prevention behavior questionnaire has two scientifically rigorous Chinese versions (26, 51). However, due to time constraints, we only chose one for the study. Future studies should use these two questionnaires to investigate and compare the differences in results.

## 5. Conclusion

This study investigated the current status of nurses' ability to prevent low back pain and its influencing factors, which could provide a reference for nursing managers to establish health promotion programs. Results showed that the nurses' ability to prevent low back pain was at a moderate level. Participation in prevention training before, perceived stress at work, and working hours per week were the influencing factors. This study has also indicated that nursing managers should organize various types of the training program, strengthen regulations to reduce nurses' workload and stress, provide a healthy workplace, and motivate nurses to prevent the occurrence of occupational low back pain.

## Data availability statement

The raw data supporting the conclusions of this article will be made available by the authors, without undue reservation.

## Ethics statement

The studies involving human participants were reviewed and approved by the Ethics Committee of Tongji Medical College, Huazhong University of Science and Technology. The patients/participants provided their written informed consent to participate in this study.

## Author contributions

YL and LG contributed to the design and supervision of the study. QL and XL contributed to data collection and analysis. QL and HL wrote the manuscript. YS and MW helped to perform the analysis with constructive discussions. All authors contributed to the article and approved the submitted version.

## References

[1] Jegnie M Afework M. Prevalence of self-reported work-related lower back pain and its associated factors in Ethiopia: a systematic review and meta-analysis. J Environ Public Health. (2021) 2021:19. 10.1155/2021/6633271 34603457 PMC8486508

[2] Coggon D Ntani G Palmer KT Felli VE Harari R Barrero LH. Disabling musculoskeletal pain in working populations: is it the job, the person, or the culture? Pain. (2013) 154:856–63. 10.1016/j.pain.2013.02.008 23688828 PMC3675684

[3] Mete Izci S Cetinkaya B. The effect of work stress, workload, and social support on nurses' self-perceptions of parenting roles during Covid-19 pandemic. J Nurs Manag. (2022) 30:4322–9. 10.1111/jonm.13838 36192809

[4] Budhrani-Shani P Berry DL Arcari P Langevin H Wayne PM. Mind-body exercises for nurses with chronic low back pain: an evidence-based review. Nurs Res Pract. (2016) (2016):9018036. 10.1155/2016/9018036 27446610 PMC4947504

[5] Van Hoof W O'Sullivan K O'Keeffe M Verschueren S O'Sullivan P Dankaerts W. The efficacy of interventions for low back pain in nurses: a systematic review. Int J Nurs Stud. (2018) 77:222–31. 10.1016/j.ijnurstu.2017.10.015 29121556

[6] Monteiro MS Alexandre NM Ilmarinen J Rodrigues CM. Work ability and musculoskeletal disorders among workers from a public health institution. Int J Occup Saf Ergon. (2009) 15:319–24. 10.1080/10803548.2009.11076813 19744373

[7] Feng CK Chen ML Mao IF. Prevalence of and risk factors for different measures of low back pain among female nursing aides in Taiwanese nursing homes. BMC Musculoskelet Disord. (2007) 8:52. 10.1186/1471-2474-8-52 17593305 PMC1920507

[8] Skela-Savic B Pesjak K Hvalic-Touzery S. Low back pain among nurses in Slovenian hospitals: cross-sectional study. Int Nurs Rev. (2017) 64:544–51. 10.1111/inr.12376 28444732

[9] Laštovková A Nakládalová M Fenclová Z Urban P. Gad'ourek P Lebeda T. Low-back pain disorders as occupational diseases in the czech republic and 22 european countries: comparison of national systems, related diagnoses and evaluation criteria. Cent Eur J Public Health. (2015) 23:244–51. 10.21101/cejph.a4185 26615658

[10] Sharma S Shrestha N Jensen MP. Pain-related factors associated with lost work days in nurses with low back pain: a cross-sectional study. Scand J Pain. (2016) 11:36–41. 10.1016/j.sjpain.2015.11.007 28850467

[11] Duffett-Leger L Beck AJ Siddons A Bright KS Alix Hayden K. What do we know about interventions to prevent low back injury and pain among nurses and nursing students? A scoping review. Can J Nurs Res. (2022) 54:392–439. 10.1177/08445621211047055 34860587

[12] Yang H-H Chung Y-C Szeto P-P Yeh M-L Lin J-G. Laser acupuncture combined with auricular acupressure improves low-back pain and quality of life in nurses: a randomized controlled trial. J Integr Med. (2023) 21:26–33. 10.1016/j.joim.2022.10.004 36402666

[13] Nkhata LA Brink Y Ernstzen D Louw QA. Nurses back pain beliefs, coping strategies and factors associated with participant activation for self-management of back pain. J Adv Nurs. (2021) 77:3772–83. 10.1111/jan.14890 34009680

[14] Roman-Liu D Kaminska J Tokarski T. Effectiveness of workplace intervention strategies in lower back pain prevention: a review. Ind Health. (2020) 58:503–19. 10.2486/indhealth.2020-0130 32968038 PMC7708737

[15] Hernandez-Lucas P Leiros-Rodriguez R Lopez-Barreiro J Garcia-Soidan JL. Is the combination of exercise therapy and health education more effective than usual medical care in the prevention of non-specific back pain? A systematic review with meta-analysis. Ann Med. (2022) 54:3107–16. 10.1080/07853890.2022.2140453 36331870 PMC9639467

[16] Driessen MT Proper KI van Tulder MW Anema JR Bongers PM van der Beek AJ. The effectiveness of physical and organisational ergonomic interventions on low back pain and neck pain: a systematic review. Occup Environ Med. (2010) (67):277–85. 10.1136/oem.2009.047548 20360197

[17] Sihawong R Janwantanakul P Jiamjarasrangsi W. A prospective, cluster-randomized controlled trial of exercise program to prevent low back pain in office workers. Eur Spine J. (2014) (23):786–93. 10.1007/s00586-014-3212-3 24492949 PMC3960439

[18] Nguyen TT Nguyen TH Hoang DL Hoang TG Pham MK. Effectiveness of interventions to prevent musculoskeletal disorders among district hospital nurses in Vietnam. Biomed Res Int. (2022) (2022):1539063. 10.1155/2022/1539063 35309175 PMC8930246

[19] Sowah D Boyko R Antle D Miller L Zakhary M Straube S. Occupational interventions for the prevention of back pain: Overview of systematic reviews. J Safety Res. (2018) 66:39–59. 10.1016/j.jsr.2018.05.007 30121110

[20] Xu GQ. Contemporary significance and development of competence-based curriculum model. J Vocat Educ. (2022) 38:57–64. 10.3969/j.issn.1001-7518.2022.1.zjlt202201007

[21] Goudreau J Pepin J Larue C Dubois S Descoteaux R Lavoie P. A competency-based approach to nurses' continuing education for clinical reasoning and leadership through reflective practice in a care situation. Nurse Educ Pract. (2015) 15:572–8. 10.1016/j.nepr.2015.10.013 26559351

[22] Alghadir AH Al-Abbad H Buragadda S Iqbal A. Influence of work-related safety and health guidelines on knowledge and prevalence of occupational back pain among rehabilitation nurses in Saudi Arabia: a 6-month follow-up study. Int J Environ Res Public Health. (2021) 18:8711. 10.3390/ijerph18168711 34444458 PMC8394852

[23] Kazemi SS Tavafian SS Hidarnia A Montazeri A. Development and validation of an instrument of occupational low back pain prevention behaviours of nurse. J Adv Nurs. (2020) 76:2747–56. 10.1111/jan.14459 32748999

[24] Yip Y A. study of work stress, patient handling activities and the risk of low back pain among nurses in Hong Kong. J Adv Nurs. (2001) 36:794–804. 10.1046/j.1365-2648.2001.02037.x 11903709

[25] Li L Cao H Yang L Yan C Wang X. and Ma Y. Risk perception and mental health among college students in China during the COVID-19 pandemic: a moderated mediation model. Front Psychiatry. (2022) 13:955093. 10.3389/fpsyt.2022.955093 35978842 PMC9376247

[26] Lai X Li Q Yi Z She C Zhang J Xu T. Reliability and validity of the Chinese version of nurses' occupational low back pain prevention behaviours questionnaire. Chin Nurs Manage. (2022) 22:38–42. 10.3969/j.issn.1672-1756.2022.01.00935400039

[27] Paulsamy P Alshahrani SH Sampayan ELE Qureshi AA Venkatesan K Sethuraj P. Knowledge and practices on risk factors and prevention of varicose vein among operation room nurses of the selected hospitals. J Pharm Res Int. (2021) 33:294–9. 10.9734/jpri/2021/v33i43B3255535024509

[28] Prajwal MS Kundury KK Sujay MJ. Assessing the awareness on occupational safety and health hazards among nursing staff of a teaching hospital. J Family Med Prim Care. (2020) 9:5961–70. 10.4103/jfmpc.jfmpc_1025_20 33681027 PMC7928124

[29] Al-Eisa E Al-Abbad H. Occupational back pain among rehabilitation nurses in Saudi Arabia: the influence of knowledge and awareness. Workplace Health Saf. (2013) 61:401–7. 10.3928/21650799-20130816-91 23957832

[30] Tian SZ Tang LM Ning WJ. Investigation on “knowledge-attitude-behavior” related to nurses occupational low back in ICU. J Nurs Train. (2013) 28:1973–6. 10.16821/j.cnki.hsjx.2013.21.024

[31] Chung MC Juang WC Li YC. Perceptions of shared decision making among health care professionals. J Eval Clin Pract. (2019) 25:1080–7. 10.1111/jep.13249 31410954

[32] Fida R Laschinger HKS Leiter MP. The protective role of self-efficacy against workplace incivility and burnout in nursing: a time-lagged study. Health Care Manage Rev. (2018) (43):21–9. 10.1097/HMR.0000000000000126 27755174

[33] Kazemi SS Tavafian SS Hiller CE Hidarnia A Montazeri A. Promoting behavior-related low back health in nurses by in-person and social media interventions in the workplace. BMC Nurs. (2022) 21:271. 10.1186/s12912-022-01045-3 36199140 PMC9535867

[34] Aboagye AK Dai B Bakpa EK. Influence of risk perception on task and contextual performance: a case of work-related musculoskeletal disorders in nurses. Eval Health Prof. (2022) 45:126–36. 10.1177/0163278720975071 33291982

[35] Yang S Li L Wang L Zeng J Li Y. Risk factors for work-related musculoskeletal disorders among intensive care unit nurses in China: a structural equation model approach. Asian Nurs Res. (2020) 14:241–8. 10.1016/j.anr.2020.08.004 32858213

[36] Gohner W Schlicht W. Preventing chronic back pain: evaluation of a theory-based cognitive-behavioural training programme for patients with subacute back pain. Patient Educ Couns. (2006) 64:87–95. 10.1016/j.pec.2005.11.018 16540279

[37] Bjornaraa J Bowers A Mino D Choice D Metz D Wagner K. Effects of a remotely delivered cognitive behavioral coaching program on the self-rated functional disability of participants with low back pain. Pain Manag Nurs. (2022) 23:397–410. 10.1016/j.pmn.2021.08.006 34706832

[38] Ross A Bevans M Brooks AT Gibbons S Wallen GR. Nurses and health-promoting behaviors: knowledge may not translate into self-care. AORN J. (2017) 105:267–75. 10.1016/j.aorn.2016.12.018 28241948 PMC5536335

[39] Otto AK Wollesen B. Multicomponent exercises to prevent and reduce back pain in elderly care nurses: a randomized controlled trial. BMC Sports Sci Med Rehabil. (2022) 14:114. 10.1186/s13102-022-00508-z 35729667 PMC9210633

[40] Ziam S Lakhal S Laroche E Lane J Alderson M Gagne C. Musculoskeletal disorder (MSD) prevention practices by nurses working in health care settings: Facilitators and barriers to implementation. Appl Ergon. (2023) (106):103895. 10.1016/j.apergo.2022.103895 36087540

[41] Dennis CL. Peer support within a health care context: a concept analysis. Int J Nurs Stud. (2003) 40:321–32. 10.1016/S0020-7489(02)00092-5 12605954

[42] Ziam S Laroche E Lakhal S Alderson M Gagne C. Application of MSD prevention practices by nursing staff working in healthcare settings. Int J Ind Ergonom. (2020) 77:102959. 10.1016/j.ergon.2020.102959

[43] Stevens ML Boyle E Hartvigsen J Mansell G Sogaard K Jorgensen MB. Mechanisms for reducing low back pain: a mediation analysis of a multifaceted intervention in workers in elderly care. Int Arch Occup Environ Health. (2019) 92:49–58. 10.1007/s00420-018-1350-3 30173369

[44] Yau SY Xiao XY Lee LY Tsang AY Wong SL Wong KF. Job stress among nurses in China. Appl Nurs Res. (2012) 25:60–4. 10.1016/j.apnr.2011.07.001 21855294

[45] National Health Commission of the People's Republic of China. Notice on Further Strengthening the Nursing Work in Medical Institutions. (2020) Available online at: http://www.gov.cn/zhengce/zhengceku/2020-09/02/content_5539428.htm (accessed November 6, 2022).

[46] Williamson L Burog W Taylor RM. A scoping review of strategies used to recruit and retain nurses in the health care workforce. J Nurs Manag. (2022) (30):2845–53. 10.1111/jonm.13786 36056545

[47] Ruotsalainen JH Verbeek JH Marine A Serra C. Preventing occupational stress in healthcare workers. Cochrane Database Syst Rev. (2015) 13:CD002892. 10.1002/14651858.CD002892.pub5 25847433 PMC6718215

[48] Denis A Zelmar A Le Pogam MA Chaleat-Valayer E Bergeret A Colin C. The PRESLO study: evaluation of a global secondary low back pain prevention program for health care personnel in a hospital setting. Multicenter Rand Interv BMC Musculoskel Dis. (2012) 13:234. 10.1186/1471-2474-13-234 23181446 PMC3579727

[49] Ayre J Jenkins H McCaffery KJ Maher CG Hancock MJ. Physiotherapists have some hesitations and unmet needs regarding delivery of exercise programs for low back pain prevention in adults: a qualitative interview study. Musculoskel Sci Prac. (2022) 62:102630. 10.1016/j.msksp.2022.102630 35932753

[50] Jaromi M Nemeth A Kranicz J Laczko T Betlehem J. Treatment and ergonomics training of work-related lower back pain and body posture problems for nurses. J Clin Nurs. (2012) 21:1776–84. 10.1111/j.1365-2702.2012.04089.x 22594388

[51] Zhang CQ Yang Z Zhang HJ. Psychometric evaluation of the chinese version of occupational lowback pain prevention behaviors questionnaire among clinical nurses: a validation study. Front Public Health. (2022) 10:827604. 10.3389/fpubh.2022.827604 35400039 PMC8984022

